# A Giant Nodule of the Soft Palate Mimicking a Tumor: A Case of Oropharyngeal Tuberculosis

**DOI:** 10.7759/cureus.31891

**Published:** 2022-11-25

**Authors:** Aboubakr Mabrouki, Azzedine lachkar, Adil Abdenbitsen, Fahd Elayoubi

**Affiliations:** 1 Otolaryngology, Head and Neck Surgery, Faculty of Medicine and Pharmacy, Mohammed VI University Hospital Centre/Mohammed I University, Oujda, MAR; 2 Otorhinology, Faculty of Medicine and Pharmacy, Mohammed VI University Hospital Centre/Mohammed I University, Oujda, MAR; 3 Maxillofacial Surgery, Faculty of Medicine and Pharmacy, Mohammed VI University Hospital Centre/Mohammed I University, Oujda, MAR

**Keywords:** histology, nodule, oropharynx, mycobacterium tuberculosis, soft palate

## Abstract

Tuberculosis is an infectious disease caused by a bacterium called *Mycobacterium tuberculosis*. According to the World Health Organization, tuberculosis is the leading cause of death by an infectious disease worldwide. We describe here a rare case of tuberculosis that presented as a giant nodule of the soft palate mimicking a tumor. A 50-year-old man was admitted to the oral and facial surgery department for odynophagia and nocturnal snoring. The clinical examination of the oral cavity revealed a mass on the right side of the soft palate, pushing back the uvula on the left, measuring 3 cm in length, nodular in appearance, hard to palpate, and painless with no inflammatory sign of the mucosa opposite. A contrast-injected cervicofacial scan and magnetic resonance imaging were requested that showed a heterogeneous mass on the right side of the soft palate. The therapeutic decision was to perform a biopsy under general anaesthesia, with a histopathological study of the mass. Intraoperatively, the mass was detachable and completely removable. The definite histopathological examination of the surgical specimen was in favour of tuberculosis of the soft palate.

## Introduction

Tuberculosis is an infectious disease caused by bacterium *Mycobacterium tuberculosis*. According to the World Health Organization, this infection is the leading cause of death because of infectious diseases worldwide, ahead of AIDS [1]. The otorhinolaryngological (or ENT) presentation of the disease is dominated by lymph node involvement. However, extra-ganglionic involvement is not exceptional and its clinical expression is related to the specific pathology of the affected organ rather than the tuberculosis disease. It can affect the oral cavity, larynx, pharynx, salivary glands, sinuses, ear, nose and the thyroid gland [2]. Several paraclinical examinations are requested when oropharyngeal tuberculosis is suspected, but histopathological examination remains the most sensitive.

## Case presentation

A 50-year-old man was admitted to the oral and facial surgery department for odynophagia and nocturnal snoring. The patient had antecedent pulmonary tuberculosis treated two years ago, and was an active smoker with 20 packs/year. He presented with a complaint of nocturnal snoring and odynophagia for the solids and then for the liquids, without fever or night sweats, of five-month duration with visualization of an endobuccal mass that was progressively increasing in size.

**Figure 1:**
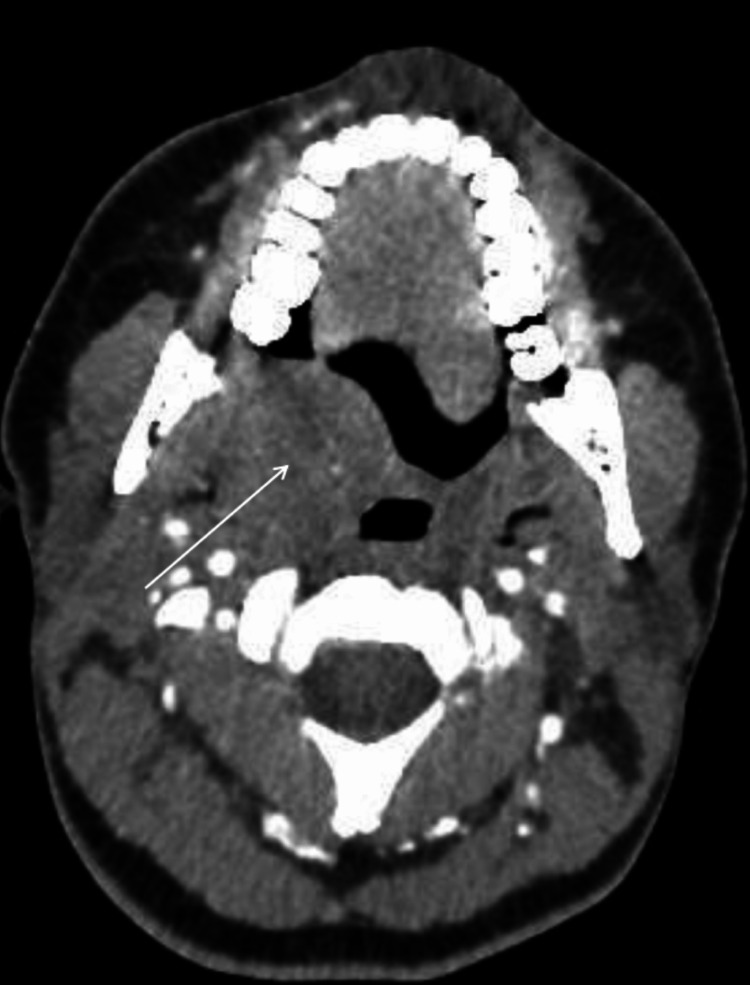
Cervicofacial scan showing an oval mass in the soft palate measuring 32 x 29 x 26 mm (arrow) with a regular contour and tissue density with the presence of microcalcification within the mass without extension to the hard palate or the homolateral tonsillar compartment

The clinical examination of the oral cavity revealed a mass on the right side of the soft palate, pushing back the uvula on the left, measuring 3 cm in length, nodular in appearance, hard to palpate, and painless with no inflammatory sign of the mucosa opposite. The homolateral tonsil was normal in appearance. The cervical examination showed some centimetric jugulocarotid adenopathies superior homolateral to the soft palate nodule. The nasofibroscopic examination revealed a bulge in the dorsal surface of the soft palate with a difference in the passage of the nasofibroscope on the right side of the mass. Chest radiography was done and the results were normal. His blood counts were normal, and Epstein-Barr virus (EBV) and human immunodeficiency virus (HIV) serology test results were negative. A contrast-injected cervicofacial scan showed an oval mass in the soft palate measuring 32 x 29 x 26 mm with a regular contour and tissue density with the presence of microcalcification within the mass without extension to the hard palate or the homolateral tonsillar compartment (Figure 1).

**Figure 2:**
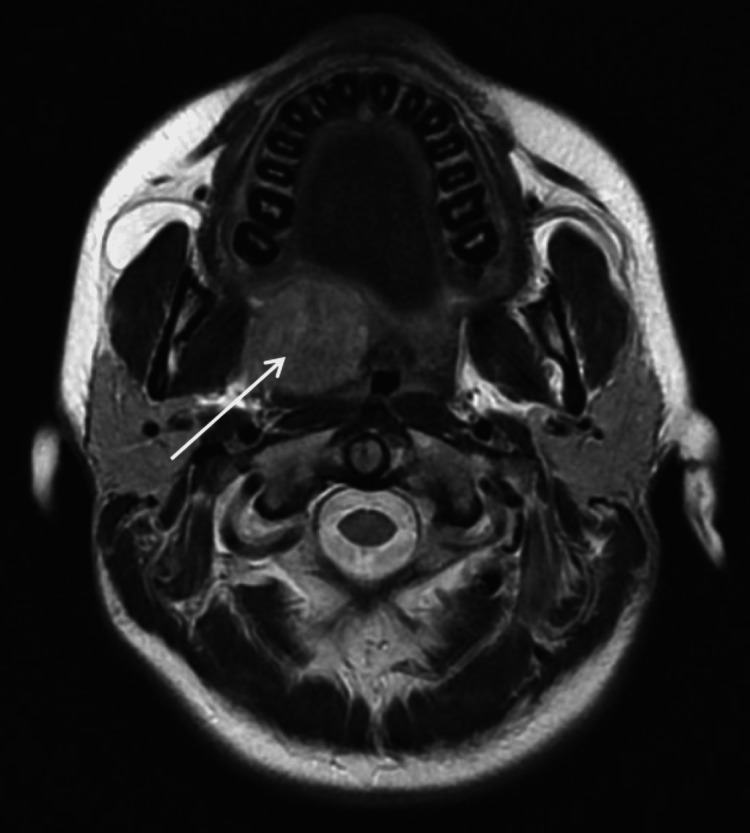
Magnetic resonance imaging showing a heterogeneous mass on the right side of the soft palate in T2 (arrow), well limited without extension to the surrounding tissues

Magnetic resonance imaging showed a heterogeneous mass on the right side of the soft palate in T2, well limited without extension to the surrounding tissues (Figure 2).

**Figure 3:**
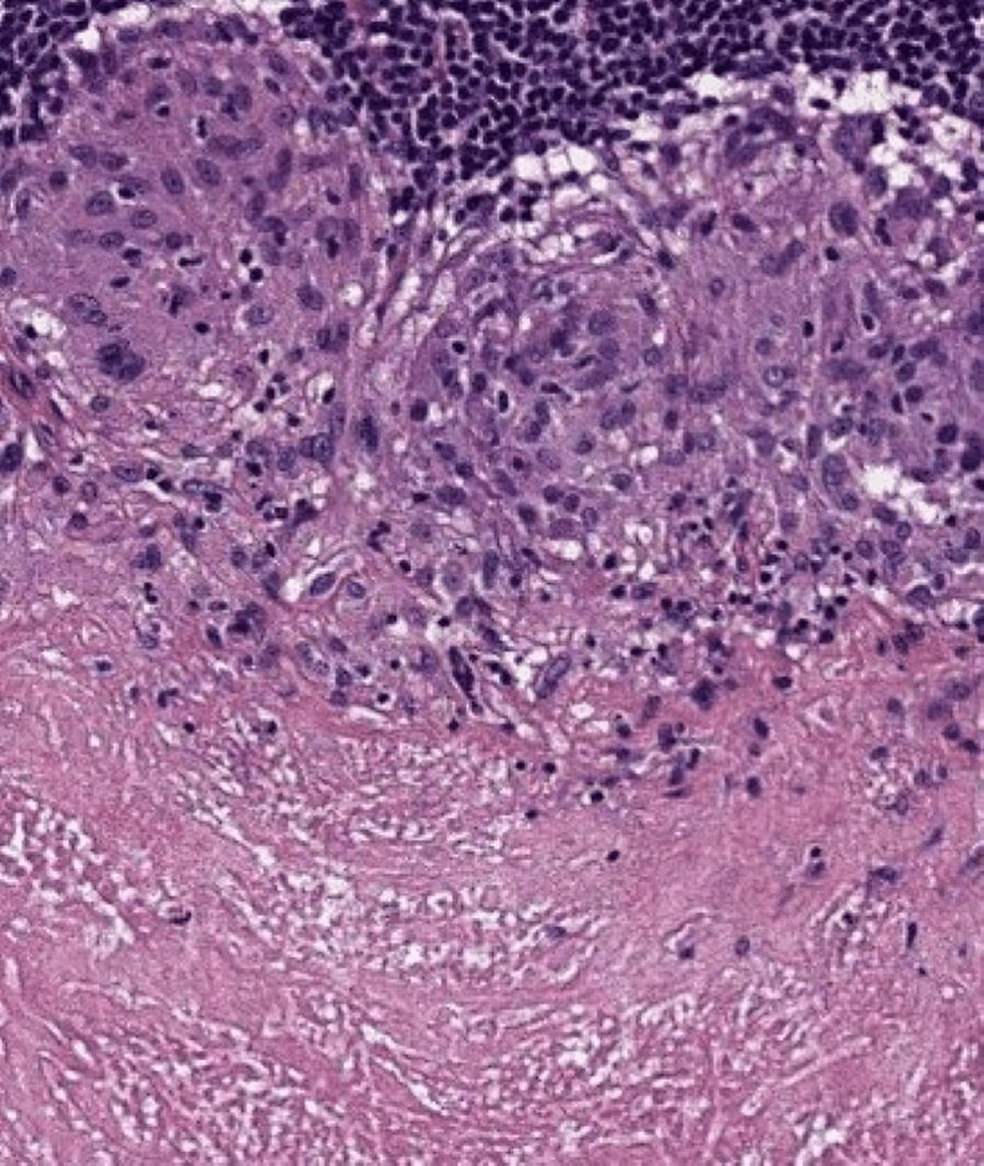
A photomicrograph showing numerous epithelioid granulomas with the presence of large amounts of caseating necrosis, and granulomas heavily surrounded by a lymphoid infiltrate (hematoxylin and eosin, 100X)

The therapeutic decision was to perform a biopsy under general anaesthesia, with a histopathological study of the mass. Intraoperatively, the mass was detachable and completely removable. The definite histopathological examination of the surgical specimen was in favour of tuberculosis of the soft palate that showed numerous epithelioid granulomas with the presence of caseating necrosis. Granulomas were heavily surrounded by a lymphoid infiltrate (Figure 3).

The patient was treated with anti-tuberculosis drugs for six months according to the Revised National Tuberculosis Control Programme (RNTCP), and the clinical examination result after treatment was normal without visualization of lesions on the soft palate.

## Discussion

Tuberculosis in ENT presentation can take on several clinical and paraclinical aspects that are not very specific, often evoking a tumoral or inflammatory pathology [3]. The localization of tuberculosis in the upper aerodigestive tract has become rare. It is secondary to dissemination by hematogenesis or direct contact of the oral mucosa with infected expectorations from a pulmonary focus [4,5]. Patients with a history of dental extractions or lacerations are at an increased risk of developing oral tuberculosis. Other factors that favour the occurrence of tuberculosis in all its forms are also involved in oral involvement, namely, low socioeconomic status, promiscuity, tuberculosis infection and immunosuppressed states such as HIV, diabetes and malnutrition [6].

Smoking is a factor that promotes the weakening of the oral mucosa and the inoculation of infectious agents such as tuberculosis. Our patient was an active smoker. Men are more predisposed than women to orpharyngeal involvement, as shown in the recent series by Gehrke et al. [7,8].

Tuberculosis of the oral cavity can present in several clinical forms such as nodule induration or fissures, but ulceration remains the most reported clinical aspect [9]. Our patient presented with an induration in the soft palate. Scans and magnetic resonance imaging are not very specific for soft palate induration. Several differential diagnoses can mimic the same clinical presentation, namely, pleomorphic adenoma, lymphoma and sarcoma. Several additional examinations are required when tuberculosis is suspected, including chest radiography, cultures and sputum smears. The histopathological examination can contribute to the confirmation of the diagnosis and is considered to be the examination with the most positive result [8]. It presents as a gigantocellular epithelioid granuloma with caseous necrosis consisting of aggregates of macrophages converted into cells, surrounded by lymphocytes and plasma cells, and aggregates of multinucleated Langerhans giant cells, whose nuclei are arranged in a horseshoe shape [10]. The type of organisation of the granulomas, their number and the presence of necrosis can define two types of lesions that will be detected on histological examination. The first type is the caseous granuloma and comprises well-organised granulomas characterised by a cluster of epithelioid histiocytes, with Langerhans giant cells, which is a typical histopathological feature of tuberculosis [11,12]. The second type includes poorly organised granulomas with a mixture of lymphocytes, histiocytes, plasma cells and Langerhans giant cells that correspond to non-caseating granulomas [12].

The RNTCP suggests a six-month course of anti-tuberculosis treatment for oropharyngeal forms, with an initial two-month treatment phase consisting of the following drugs: rifampicin, or R (10 mg/kg body weight), isoniazid, or H (5 mg/kg body weight), pyrazinamide, or Z (25 mg/kg body weight) and ethambutol, or E (15 mg/kg body weight). This is followed by a second phase of four months that includes the administration of isoniazid and rifampicin at the same dosage as the first phase. Our patient received a therapeutic schema of 4RHZE and 2RH.

## Conclusions

Tuberculosis is a chronic mycobacterial infection that primarily affects the respiratory tract; when it is not ganglionic, it can affect other sites of the ENT sphere. Tuberculosis in the oropharynx remains rare. Diagnosis is often difficult and delayed because the clinical and radiological signs are not specific. However, in endemic areas, the clinician should be aware of different clinical aspects and deduce the diagnosis of oropharyngeal tuberculosis.
